# Two new species of Entomobryidae (Collembola) of Taibai Mountain from China

**DOI:** 10.3897/zookeys.338.5723

**Published:** 2013-10-02

**Authors:** Xiang-Qun Yuan, Zhi-Xiang Pan

**Affiliations:** 1 Key Laboratory of Plant Protection Resources and Pest Management of Ministry Education, Entomological Museum, Northwest A&F University, Yangling, Shaanxi 712100, China; 2 School of Life Sciences, Taizhou University, Taizhou, Zhejiang 318000, China

**Keywords:** Entomobryinae, new species, chaetotaxy, Qinling

## Abstract

Taibai Mountain is the highest peak of Qinling Mountain Ridge, a climate and geographical demarcation of the southern and northern China. Collembolan species of family Entomobryidae are reported from this region for the first time in this paper. Two new species, *Homidia taibaiensis* **sp. n.** and *Sinella triseta* **sp. n**. of Entomobryinae are described. Illustrations and differences with similar species are provided.

## Introduction

Entomobryidae is the largest family of Collembola with 1736 species recorded worldwide (Bellinger et al. 1996‒2013). Among them, 60 and 64 species belong to the genera *Homidia* and *Sinella*, respectively. The two generaare affiliated to Entomobryinae, without scales on body and abundant mac (Chen and Christiansen 1993; Pan et al. 2012). The genus *Homidia* is characterized by spines present on the inner edge of dentes and “eyebrow” mac on anterior Abd. IV in adults, 8+8 ommatidia, mucro bidentate with subapical tooth larger than apical one, and mostly with a significant colour pattern (Pan et al. 2011). *Sinella* is characterized by reduced ommatidia number and pigment, bidentate mucro and without apical bulb on Ant. IV (Brook 1882).

Qinling is the east-west axial ridge, forming a natural climate and geographical barrier between the southern and northern China. Taibai Mountain is the highest peak along this ridge, located in Baoji City, Shaanxi Province, with a peak rising up to 3767.2 m. Before our study, there was no Entomobryinae species first reported from Taibai Mountain. Hear, two new species of this group are described.

## Materials and methods

Specimens were cleared in lactic acid, mounted under a coverslip in Marc André II solution, and observed using Nikon 80i microscope with phase contrast. Photographs were taken with a Nikon SMZ1000 stereomicroscope mounted with a Nikon DS-Fi1 camera. Illustrations were completed to photographs using Photoshop CS2 (Adobe Inc.). All length data were measured with NIS-Elements Documentation 3.1 software (Nikon). Cephalic dorsal chaetotaxy for the genera *Homidia* and *Sinella* were designated following Szeptycki’s (1973) and Chen and Christiansen’s system (1993), respectively, with labial palp chaetae after Fjellberg (1998), labial chaetae after Gisin (1964), dorsal chaetotaxy of terga after Szeptycki (1979).

Abbreviations: Ant. – antennal segment; Th. – thoracic segment; Abd. – abdominal segment; ms – Specialized microchaeta(e); s – Specialized ordinary chaeta(e); mac – macrochaeta(e); mic – microchaeta(e).

## Taxonomy

### Homidia taibaiensis sp. n.

http://zoobank.org/D764D6D5-81D2-4461-97F3-78A2ED1C89B2

http://species-id.net/wiki/Homidia_taibaiensis

Figures 1 –21

#### Holotype.

1♀ on slide, Baoji City, Mei County, Haoping Temple manage department, Shaanxi Province, CHINA, 34°05.67'N, 107°42.40'E, sample number S4333, collected by Xiang-Qun Yuan and Zhi-Xiang Pan, 13.VII.2012.

#### Paratypes.

2♀ on slide and 3 in alcohol, same data as holotype, all types deposited in School of Life Sciences, Taizhou University.

#### Etymology.

Named after the type locality.

#### Description.

Body length up to 2.95 mm.

**Figures 1–3.**
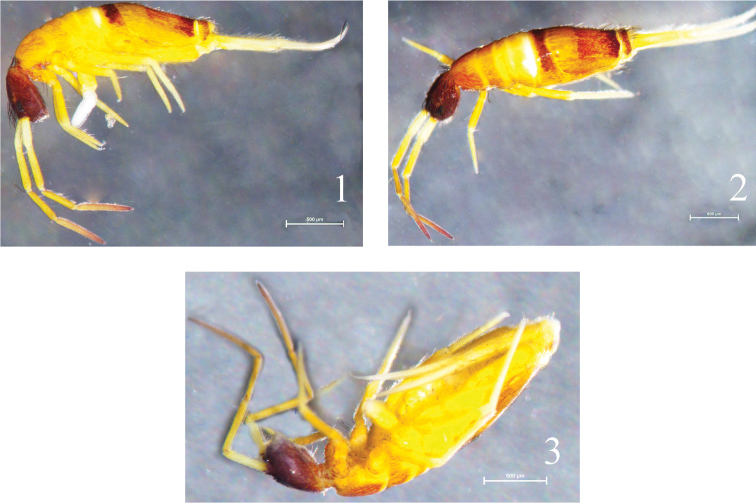
Habitus of *Homidia taibaiensis* sp. n. **1** lateral view **2** dorsal view **3** ventral view.

Colour pattern. Ground colour yellow in alcohol, including ventral side; ommatidium patches dark blue; whole head dark brown; antennae yellow except Ant. IV with slight brown pigment and gradually deeper from base to tip; dorsal side of Th. II to Abd. II with slight brown pigment and gradually lighter, posterior Abd. II with a middle and narrow white band close to Abd. III; Abd. III and Abd. V dark brown except bilaterally; Abd. IV with brown pigment and gradually deeper from anterior to posterior edge; coxae of fore and mid leg with slight brown pigment; ventral tube and furcula also yellow (Figs 1–3).

**Figures 4–10.**
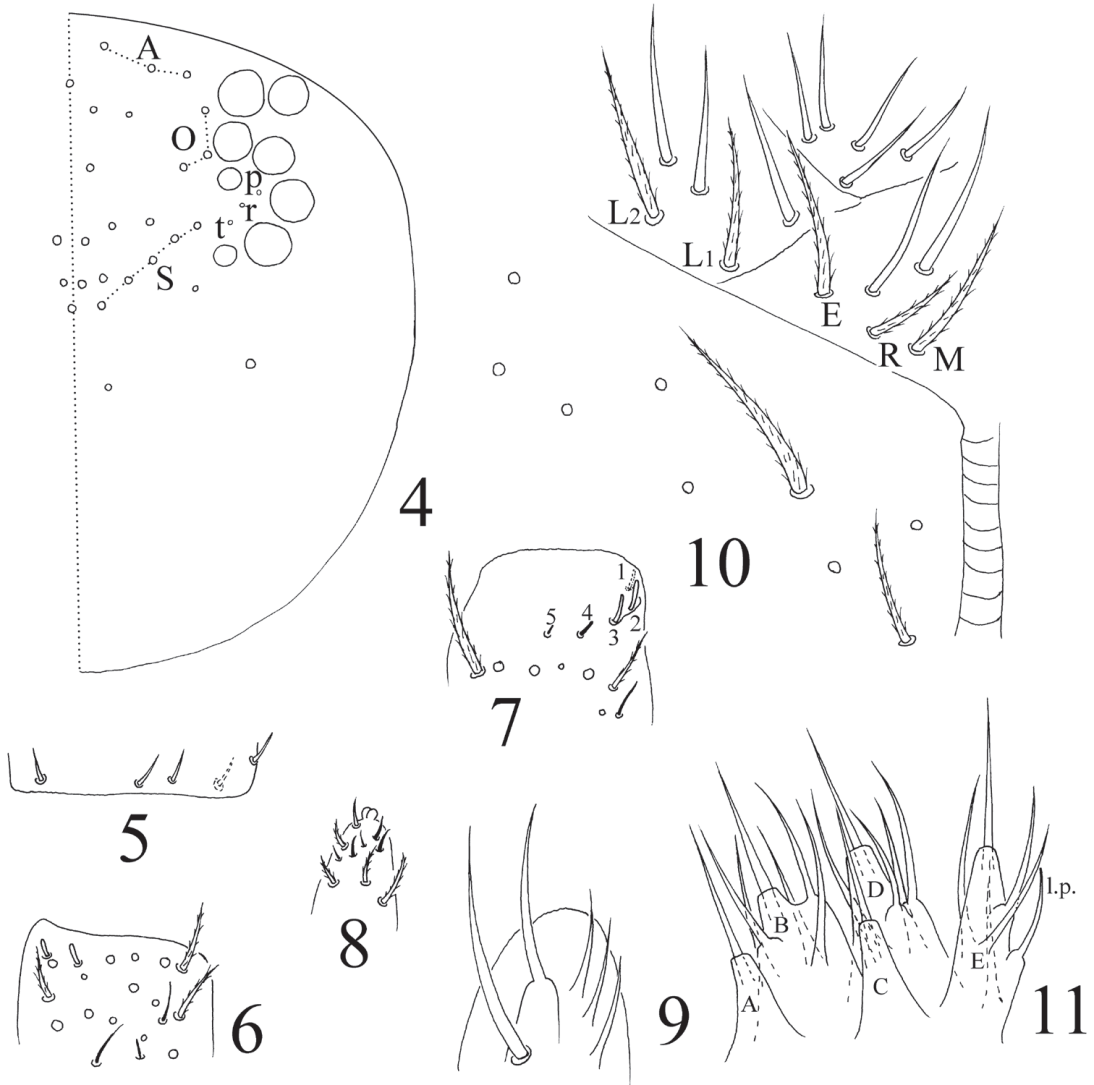
*Homidia taibaiensis* sp. n. **4** dorsal cephalic chaetotaxy **5** basal spiny chaetae of Ant. II **6** distal Ant. II **7** Ant. III organ **8** apical bulb of Ant. IV **9** maxillary outer lobe **10** labial base **11** labial palp.

Head. Ommatidia 8+8, G and H smaller than others and always difficult to observe under light microscope; interocular chaetae as p, r, t, chaeta p largest (Fig. 4). Antenna 2.05–3.31 times as long as cephalic diagonal; antennal segments ratio as I: II: III: IV = 1: 1.24–1.97: 0.91–1.52: 1.71–2.46. Ant. I base with 3 dorsal spiny chaetae, ventral side unclear; Ant. II with 2 dorsal, 3 ventral basal smooth chaetae (Fig. 5), 2–5 distal rod-like S-chaetae (Fig. 6); Ant. III organ with 2 rod-like and 3 short guard S-chaetae (Fig. 7); apical bulb of Ant. IV bilobed (Fig. 8). Dorsal cephalic chaetotaxy with 3 antennal (A), 3 ocellar (O) and 5 sutural (S) mac, posterior cephalic chaetotaxy unclear (Fig. 4). Prelabral and labral chaetae as 4/5, 5, 4, all smooth, without labral papillae. Maxillary outer lobe with 1 apical, 1 subapical chaetae and 3 sublobal hairs on sublobal plate, subapical chaeta slightly larger than apical one (Fig. 9). Proximal with 5 smooth chaetae. Chaetal formula of labial base as MREL_1_L_2_, all ciliate (Fig. 10). Five papillae A–E on labial palp with 0, 5, 0, 4, 4 guard chaetae, respectively. Lateral process (l.p.) with tip not reaching apex of papilla E (Fig. 11). Mandible with 4/5 (left/right side) teeth.

**Figures 12–14.**
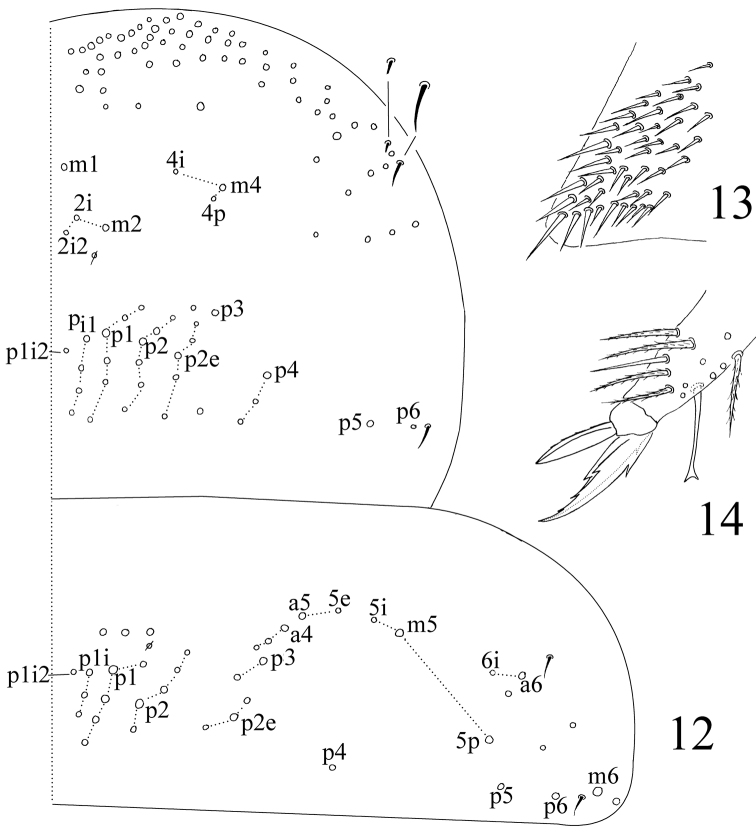
*Homidia taibaiensis* sp. n. **12** dorsal chaetotaxy of Th. II–III **13** trochanteral organ **14** apical tibiotarsus and claw of hind leg.

Thorax. Complete body s as 22/122 (Abd. IV unclear) 3; ms as 10/10100. Th. II with 4 (m1, m2, m2i and m2i2) medio-medial, 3 (m4, m4i and m4p) medio-sublateral and 3 S-chaetae (ms antero-internal to s); posterior with 26–30 mac; p4, p4i, p4i2 and p5 as mac, p6 as mic. Th. III with about 40 mac and 2 S-chaetae; p4 as mac (Fig. 12). Coxal macrochaetal formula as 3 (2 p)/4+1, 3 (3 p)/4+2 (number of pseudopores unclear). Trochanteral organ with 40–45 smooth chaetae (Fig. 13). Tenent hair clavate and slightly shorter than inner edge of unguis in length. Distal smooth chaeta on hind leg subequal to tenent hair in length. Unguis with 4 inner, 2 lateral and 1 outer teeth. Unguiculus lanceolate with outer edge serrated (Fig. 14).

**Figures 15–17.**
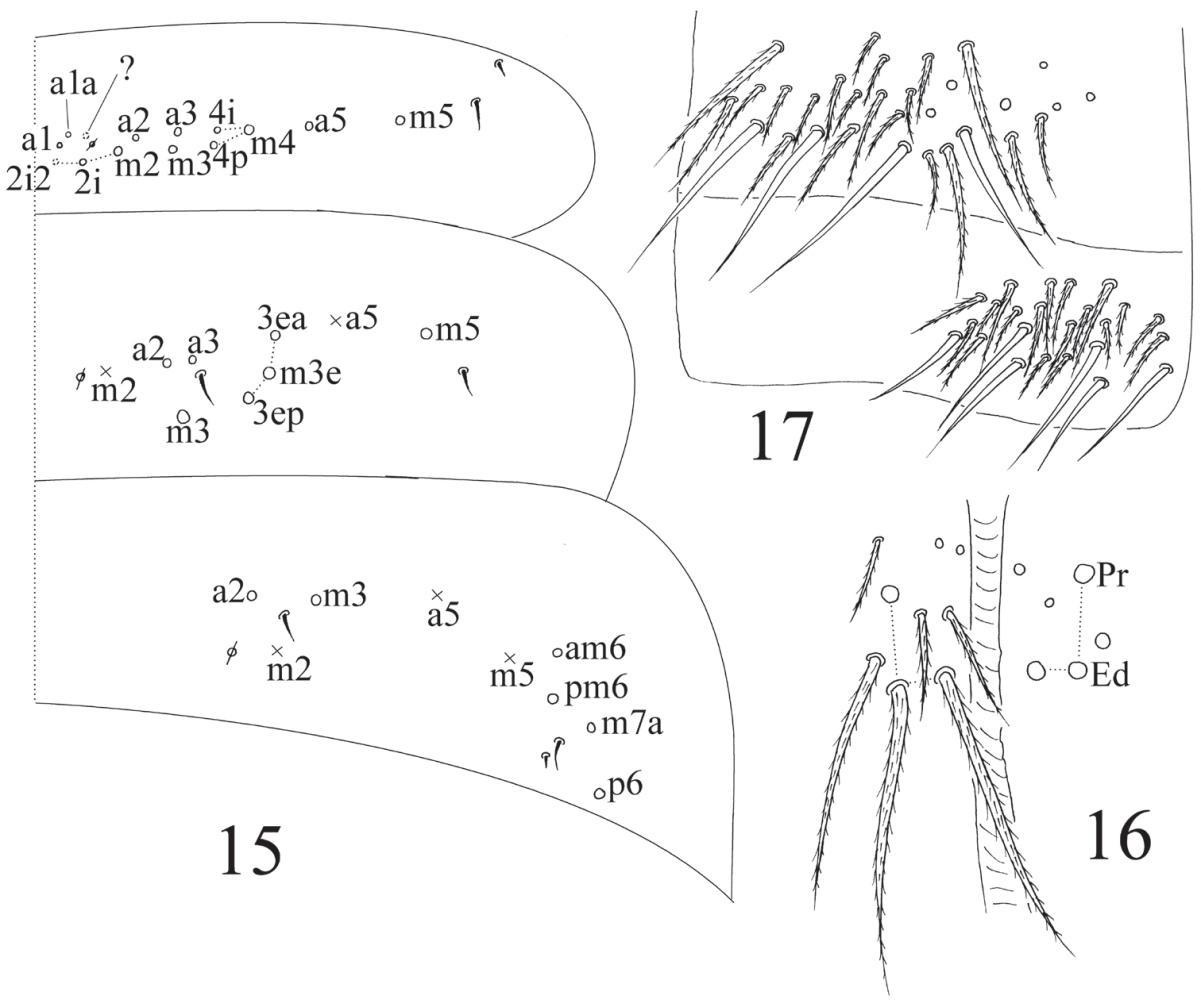
*Homidia taibaiensis* sp. n. **15** dorsal chaetotaxy of Abd. I–III **16** anterior face of ventral tube **17** posterior face and lateral flap of ventral tube.

**Figures 18–21.**
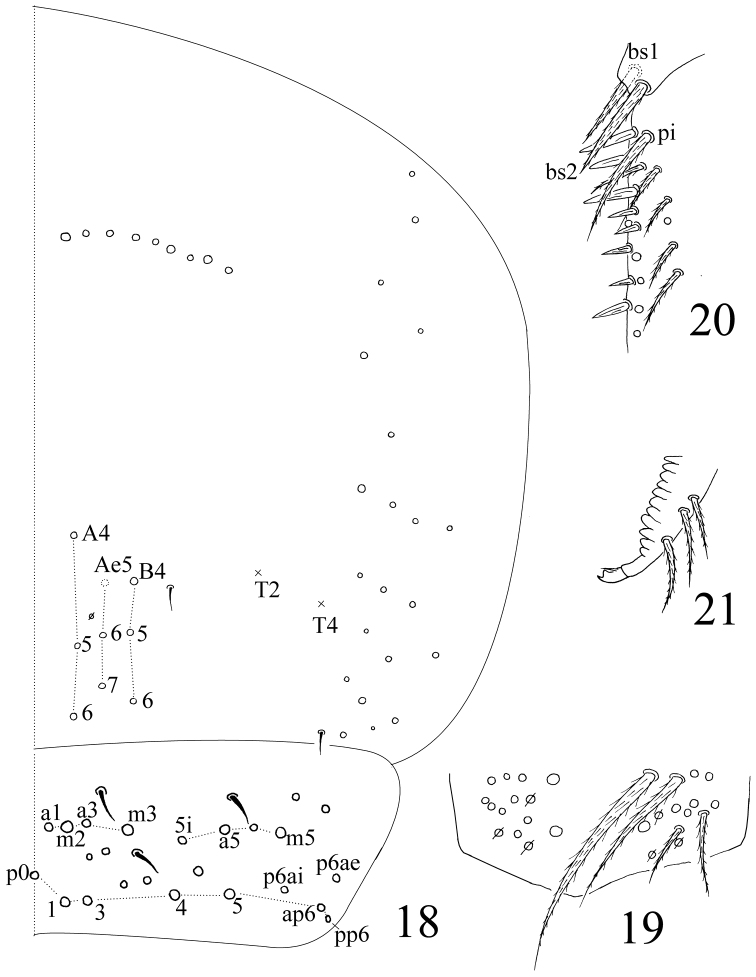
*Homidia taibaiensis* sp. n. **18** dorsal chaetotaxy of Abd. IV–V **19** manubrial plaque **20** basal dentes **21** apical dentes and mucro.

Abdomen. Abd. IV as 6–11 times as Abd. III in dorsal axial length. Abd. I with 12–14 (a1–3, a1a, a5, m2–4, m2i, m4i, m4p and m5; one unclear homological mac near to pseudopore and m2i2 sometimes absent) mac and 2 S-chaetae (ms antero-external to s). Abd. II with 6 (a2, a3, m3, m3e, m3ea and m3ep) central, 1 (m5) lateral mac and 2 S-chaetae. Abd. III with 2 (a2 and m3) central, 4 (am6, pm6, p6 and m7a) lateral mac and 3 S-chaetae (Fig. 15). Abd. IV with many (precise number unclear) elongate and 2 (as and ps) short S-chaetae; “eyebrow” with 8–10 mac arranged in irregular transverse row; posterior central with 8–9 (A4–6, Ae6, Ae7, B4–6; Ae5 sometimes absent). Abd. V with 3 S-chaetae; m3a as mic and a5i as mac (Fig. 18). Anterior face of ventral tube (VT) with many ciliate chaetae, 3+3 of them as mac, line connecting proximal (Pr) and external-distal (Ed) mac parallel to median furrow (Fig. 16); posterior face with 3 or 4 (1+1+1 or 2+2) subapical smooth chaetae; lateral flap with 6 smooth and 16–18 ciliate chaetae on each side (Fig. 17). Manubrial plaque with 3 pseudopores and 8–11 ciliate chaetae (Fig. 19). Dentes with 32–49 spines; basal chaetae (bs1 and bs2) spiny, bs1 shorter than bs2; pi ciliated and slightly longer than bs2 (Fig. 20). Mucro bidentate with subapical tooth larger than apical one; basal spine short, with tip not reaching apical tooth; distal smooth part of dentes subequal to mucro in length (Fig. 21). Tenaculum with 4+4 teeth and 1 large, multi-laterally basal ciliate chaeta.

#### Ecology.

Found in the leaf litter of *Brassica campestris* L. on farmland, altitude 1165±8 m.

#### Remarks.

This new species is characterized by unique colour pattern, such as ground colour yellow (especially VT and furcula), dark brown head (including ventral side), Abd. II with posterior white band, labial basal chaetae E and L_1_ ciliate, m5 as mac on Abd. I and 8 mac present on median posterior Abd. IV.

The new species is closest to *Homidia socia* Denis, 1929 in chaetotaxy, relative to the ciliate labial base chaetae E and L_1_, m5 on lateral Abd. I as mac, A4–6 and B4–6 as mac on posterior Abd. IV. However, it can be easily discriminated from the latter by colour pattern (without longitudinal stripe in the former, three stripes in the latter) and other characters, such as 3 mac in S sets on dorsal head (4 in the latter), m3 as mac on middle Abd. III (as mic in the latter) and without mac anterior to “eyebrow” on Abd. IV (with 2 mac in the latter). Also, this species is similar to *Homidia similis* Szeptycki, 1973 in chaetotaxy, detailed differences between them are listed in Table 1.

**Table 1.** Differences between *Homidia taibaiensis* sp. n. and other two closest species of *Homidia*.

**Characters**	***Homidia taibaiensis* sp. n.**	***Homidia socia***	***Homidia similis***
Dark longitudinal stripes from head to Abd. III	-	+	-
Whole head brown	yes	no	no
Length ratio of Abd. IV/III	6–11	<4^b^	>4^b^
Morphology of chaeta E on labial base	ciliate	ciliate	smooth
Labral papillae	0	4^b^	4
Antennal mac on dorsal head	3	4^b^	3
Mac in “eyebrow” of anterior Abd. IV	8–10	7^b^	7–9^a^
Mac on manubrial plate	8–11	9–13^a^	8^b^
Chaetae on lateral flap of ventral tube			
smooth chaetae	6	5–6^a^	5^a^
ciliate chaetae	16–18	12–24^a^	8–12^a^
Smooth chaetae on posterior of ventral tube	3 or 4	2^a^	4 or 5^a^
Relative position of ms/s on lateral Th. II	antero-internal	antero-internal^a^	antero-external^a^
Mac m5 on Abd. I	+	+^a^	-^a^
Mac of Abd. IV			
anterior to “eyebrow”	-	+^a^	-^a^
A4a	-	+^a^	-^a^
A6e	+	-^a^	+^a^
Distribution	China	China, Japan, Vietnam^b^	China^a^, Korea

Notes:

a: based on author’s observation;

b: based on Jordana’s description (2012);

+: present;

-: absent.

### Sinella triseta sp. n.

http://zoobank.org/13C71E41-EA08-40ED-84E0-8731E64DAEB0

http://species-id.net/wiki/Sinella_triseta

Figures 22 –40

#### Holotype.

1♀ on slide, Baoji City, Mei County, Haoping Temple manage department, Shaanxi Province, CHINA, 34°05.18'N, 107°42.08'E, sample number S4325, collected by Xiang-Qun Yuan, Zhi-Xiang Pan, 11.VII.2012.

#### Paratypes.

5♀, 1♂ on slide and 3 in alcohol, same data as holotype, all types deposited in School of Life Sciences, Taizhou University.

#### Etymology.

Named using the Latin words “tri+seta” (three mac on each side of posterior Abd. IV).

#### Description.

**Figure 22.**
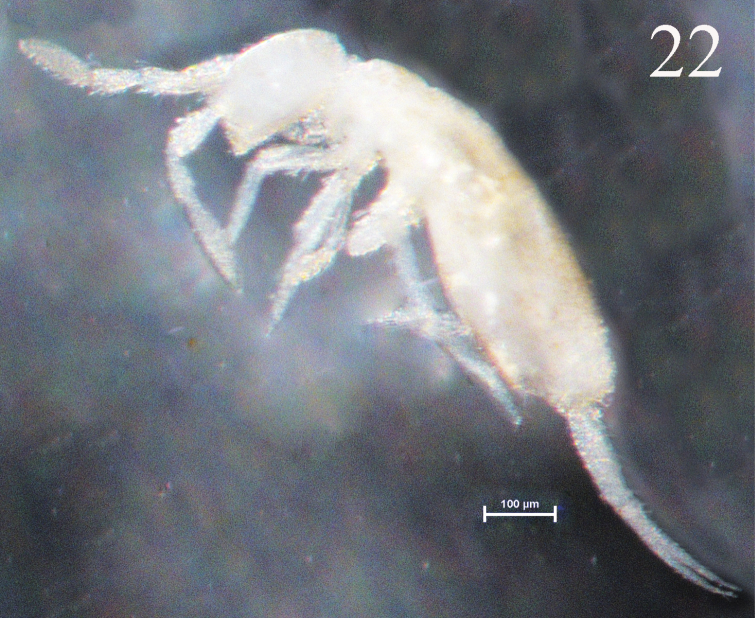
Habitus of *Sinella triseta* sp. n.

Body length up to 1.17 mm, white (Fig. 22).

**Figures 23–29.**
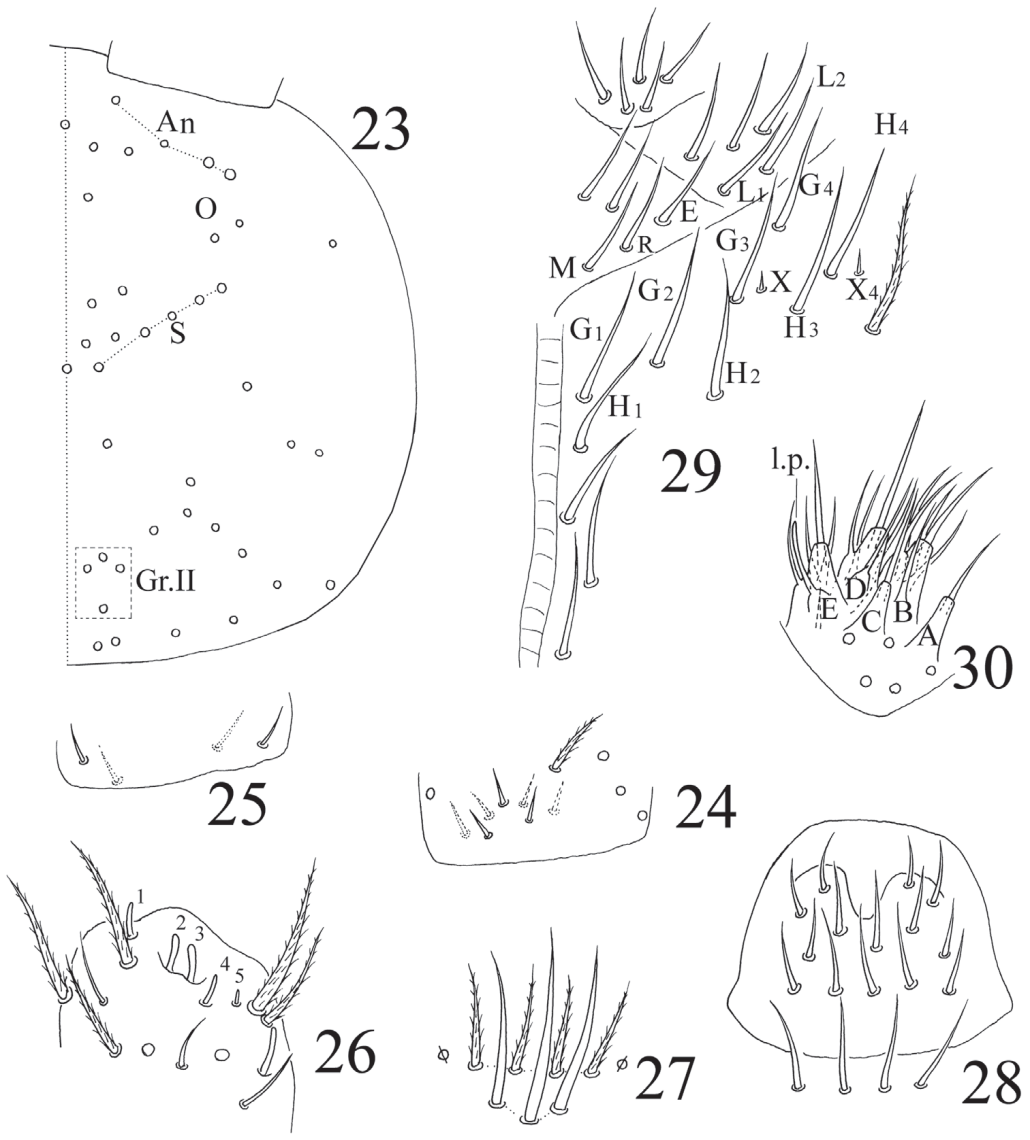
*Sinella triseta* sp. n. **23** dorsal cephalic chaetotaxy **24** basal chaetae of Ant. I **25** basal chaetae of Ant. II **26** Ant. III organ **27** clypeus **28** labrum **29** labial base **30** labial palp.

Head. Antenna 1.27–1.61 times as long as cephalic diagonal. Antennal segments ratio as I: II: III: IV = 1: 1.67–2.05: 1.42–1.71: 1.95–3.28. Smooth spiny mic at base of antennae as 3 dorsal, 4 ventral on Ant. I (Fig. 24) and 4 on Ant. II (Fig. 25). Ant. III organ with 5 rod-like S-chaetae (Fig. 26). Ommatidia absent. Dorsal cephalic chaetotaxy with 4 antennal (An), 5 sutural (S) and 4 mac in Gr. II (Fig. 23). Clypeus with 7 chaetae arranged in two lines (4 ciliate and 3 smooth) (Fig. 27). Labral papillae absent; prelabral and labral chaetae as 4/5, 5, 4, all smooth; labial intrusion U-shaped (Fig. 28). Subapical chaeta of maxillary outer lobe shorter than apical one; 3 smooth sublobal hairs on maxillary outer lobe. Labial chaetae as MREL_1_L_2_, all smooth; chaeta R subequal to M; chaetae X and X_4_ as peg-like, smooth mic; chaetae X_2_ and X_3_ absent (Fig. 29). Five papillae A–E on labial palp with 0, 5, 0, 4, 4 guard chaetae, respectively. Lateral process (l.p.) of labial palp as thick as normal chaetae, with tip beyond apex of labial papilla E (Fig. 30). Mandible with 4/5 (left/right side) teeth.

**Figures 31–34.**
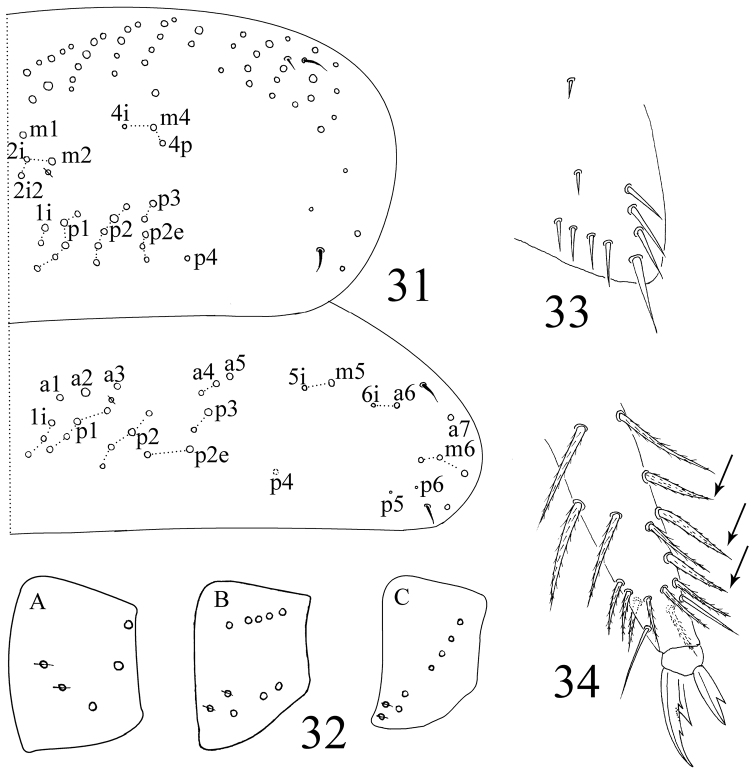
*Sinella triseta* sp. n. **31** dorsal chaetotaxy of Th. II–III **32** coxal mac formula (**A** fore leg; **B** mid leg; **C** hind leg) **33** trochanteral organ **34** tip tibiotarsus and claw of hind leg.

Thorax. Complete body s as 22/122 (14–16) 3; ms as 10/10000. Th. II with 4 (m1, m2, m2i and m2i2) medio-medial, 3 medio-lateral (m4, m4i, m4p), 18–21 posterior mac and 3 S-chaetae (ms internal to s); p4 as mac, p5 and p6 as mic, p1i2 and p4i sometimes absent. Th. III with about 30 mac and 2 lateral S-chaetae; p5, p6 and a5e as mic, p4 rarely as mac (Fig. 31). Coxal macrochaetal formula as 3 (2 p)/4+1, 3 (2 p)/4+2 (2 p) (Fig. 32). Trochanteral organ with 8–12 smooth spiny chaetae; 5–11 in arms and 2–3 between them (Fig. 33). 3–4 inner differentiated tibiotarsal chaetae “smooth” with ciliations closely appressed to axis. Tenent hair all acuminate and subequal to inner edge of unguis. Unguis with 3 inner teeth, basal paired teeth unequal, outer one larger. Unguiculus acuminate with a large tooth on outer edge (Fig. 34).

**Figure 35.**
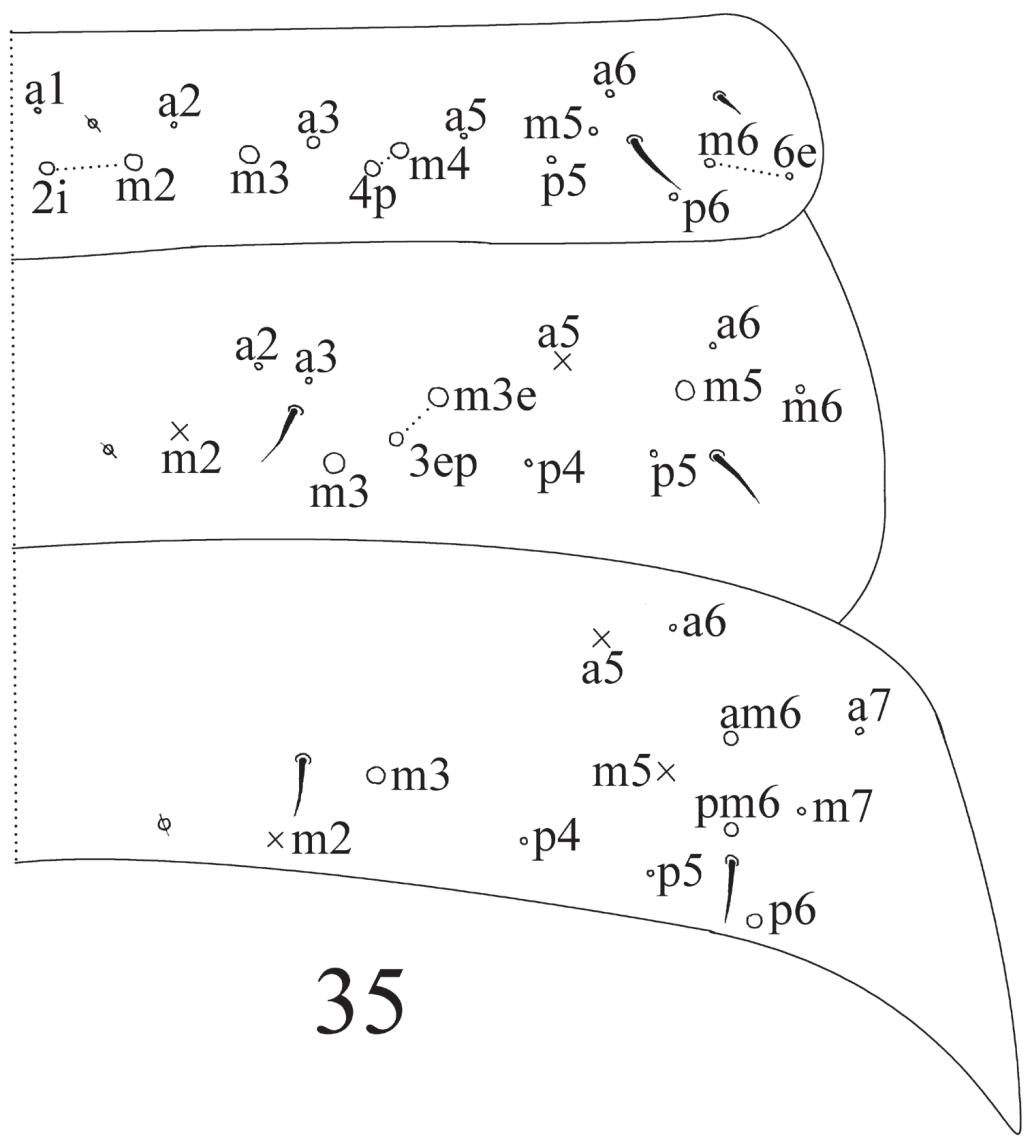
dorsal chaetotaxy of Abd. I–III of *Sinella triseta* sp. n.

**Figures 36–40.**
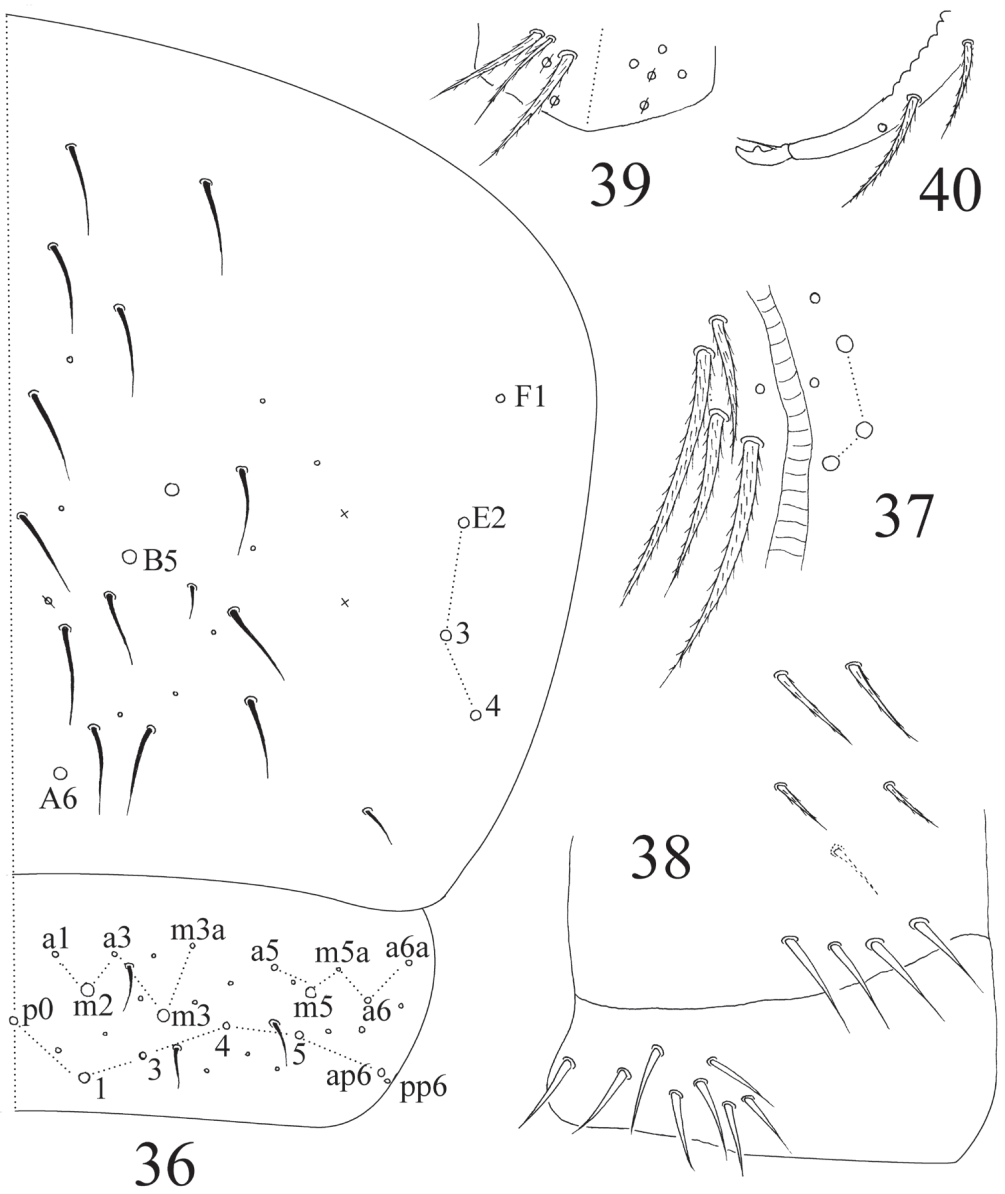
*Sinella triseta* sp. n. **36** dorsal chaetotaxy of Abd. IV–V **37** anterior face of VT **38** posterior face and lateral flap of VT **39** manubrial plaque **40** apical dentes and mucro.

Abdomen. Abd. IV 2.94–4.34 times as Abd. III in dorsal axial length. Abd. I with 6 (a3, m2–4, m2i, m4p) mac and 2 S-chaetae (ms antero-external to s). Abd. II with 3 (m3, m3e, m3ep) central, 1 (m5) lateral mac and 2 S-chaetae. Abd. III with 1 (m3) central, 3 (am6, pm6, p6) lateral mac and 2 S-chaetae (lateral ms absent) (Fig. 35). Abd. IV with 3 central (A6, B5 and anterior one homology uncleared mac), 4 lateral mac (F1, E2–4), 12–14 elongate and 2 normal S-chaetae. Abd. V with 3 obvious mac (m2, m3 and m5) and 3 S-chaetae (Fig. 36). Tenaculum with 4+4 teeth and one large basal chaeta. Anterior face of ventral tube with 5+5 ciliate chaetae (Fig. 37); posterior with 2+2 basal weekly ciliate and 2+2 subapical smooth chaetae, an additional smooth chaeta sometimes present between basal and apical region; lateral flap with 7–8 smooth chaetae, among them 0–2 weekly ciliated among them (usually unclear under light microscope) (Fig. 38). Manubrium without smooth chaetae. Manubrial plaque with 2 pseudopores and 3 ciliate chaetae (Fig. 39). Distal smooth part of dentes approximately 2 times as mucro in length. Mucro bidentate with basal spine long with tip reaching apex of apical tooth (Fig. 40).

#### Ecology.

Found under stones in forest, altitude 1185±10 m.

#### Remarks.

This new species is characterized by 3 mac on posterior middle Abd. IV, ommatidia absent, labial base chaeta R subequal to M in length, X_2_ and X_3_ on ventral side of head absent, Abd. I–III with 6, 4, 4 mac, respectively, and clypeus with 7 mac arranged in two lines.

This species is most similar to *Sinella yunnanica* Zhang & Deharveng, 2011 in colour pattern, without ommatidia, claw, mucro, lateral process of labial palp, manubrial plaque and chaetotaxy of head and Th. II–Abd. III. However, it differ from latter in labial base chaeta R subequal to M (obviously short in the latter), X_2_ absent (present in the latter), 3 central mac on Abd. IV (5 in the latter), ventral tube with 5+5 mac on anterior face (about 8 in the latter) and 8–9 smooth chaetae on posterior face (12 in the latter). Also, this new species is similar to *Sinella colorata* Zhang et al. 2010 and *Sinella pauciseta* Qu et al. 2010 in 3 mac on posterior median Abd. IV, detailed differences between them are listed in Table 2.

**Table 2.** Differences between *Sinella triseta* sp. n. and other three similar species of *Sinella*.

**Characters**	***Sinella triseta* sp. n.**	***Sinella yunnanica***	***Sinella colorata***	***Sinella pauciseta***
Colour pattern	white	white	beige-violet to pale orange	white
Number of ommatidia	0+0	0+0	3+3	1+1
Chaetae on ventral side of head				
ratio of R/M	≈1.0	0.15–0.20	≈0.50	≈0.54
X	smooth mic	smooth mic	smooth mac	ciliate mac
X4	smooth mic	smooth mic	ciliate mac	ciliate mac
Inner teeth of unguis	3	3	3	2
Tenent hair	acuminate	clavate	acuminate	acuminate
Ventral tube				
posterior smooth chaetae	8	10–12	6–10	8
smooth chaetae on lateral flap	7–8	7–10	5	6
Manubrial plaque				
pseudopores	2	2	2	?
ciliate chaetae	3	3	2	?
Mucronal basal spine	II	II	I	I
Mac of Th. II				
m2i2	+	-	-	-
p4i	-	+	- (+)	-
Mac a3 of Abd. I	+	+	-	+
Mac of Abd. II				
a2	-	-	+	-
m3ep	+	+	-	+
Chaetae of Abd. III				
mac am6 on lateral	+	+	-	+
ms	-	+	+	?
Mac on Abd. IV				
A3	-	+	-	-
B4	-	+	-	-
E2p	-	+	+	+
D3	-	-	+	+

Notes:

?: unclear characters;

I: mucronal basal spine reaching apex of subapical tooth;

II: mucronal basal spine reaching apex of apical tooth;

-: absent;

+: present.

## Supplementary Material

XML Treatment for Homidia taibaiensis

XML Treatment for Sinella triseta
